# Case Report: Remote magnetic navigation and accessory pathways ablation in a single ventricle young adult with complex corrective surgeries

**DOI:** 10.3389/fped.2024.1358505

**Published:** 2024-02-16

**Authors:** Steliana Cosmina Paja, Viviana Gondoș, Silvia Deaconu, Eliza Cinteză, Radu Vătășescu

**Affiliations:** ^1^ Cardiology Department, Clinic Emergency Hospital, Bucharest, Romania; ^2^ Department of Medical Electronics and Informatics, Polytechnic University of Bucharest, Bucharest, Romania; ^3^ ARES Centers, Bucharest, Romania; ^4^ Department of Pediatric Cardiology, “Marie Curie” Emergency Children’s Hospital, Bucharest, Romania; ^5^ 4th Department — Cardio-Thoracic Pathology, “Carol Davila” University of Medicine and Pharmacy, Bucharest, Romania

**Keywords:** case report, congenital heart disease, surgical correction procedures, accessory pathways, atrioventricular reentrant tachycardia, remote magnetic navigation, electroanatomic mapping

## Abstract

Supraventricular arrhythmias have become an increasingly significant contributor to the risk of mortality and morbidity in adults with complex congenital heart disease (CHD), especially in light of recent advances in palliative corrective surgeries. Because of their unique characteristics, they demand specific treatment approaches. While pharmaco-logical interventions are an option, they have limited effectiveness and may lead to side effects. Although performing radiofrequency ablation (RFA) can be exceptionally challenging in patients with complex CHD, due to particular vascular access and also modified anatomy, it has paved the way to enhance comprehension of the underlying mechanisms of supraventricular arrhythmias. This, in turn, enables the provision of improved therapies and, ultimately, an enhancement in the quality of life and symptom management for these patients. The purpose of this case report is to highlight the benefits of utilizing advanced technologies such as three-dimensional electro-anatomical mapping systems, remote magnetic navigation, and highly flexible mapping and ablation catheters during RFA in a young adult with complex congenital heart disease. Although he lacked venous connections to the right atrium (RA) due to multiple corrective surgeries we, remarkably, were capable to advance a decapolar deflectable diagnostic catheter inside the Fontan tunnel and from there to record and stimulate the RA. Successful ablation of two accessory pathways was achieved with no arrhythmia recurrence during follow-up.

## 1 Introduction

Supraventricular tachyarrhythmias (SVT) stand as a prevalent and challenging complication encountered in the CHD adult population. The prevalence of these tachyarrhythmias is notably influenced by various factors, including the patient's age, the specific underlying congenital defect, the type of surgical corrections previously undertaken, and the timing of those surgical interventions. With advancing age, the burden of SVT tends to increase, underscoring the complex interplay between cardiac anomalies and the aging process in CHD patients. These arrhythmias hold a notable position as the leading cause of hospital admissions among this population, primarily due to their capacity to induce debilitating symptoms, exacerbate heart failure, and escalate the risk of cerebrovascular stroke (1).

Moreover, the risk linked with SVT is compounded by the potential for rapid ventricular conduction, which can give rise to a heightened vulnerability to sudden cardiac death (2). Thus, the effective management approached of SVT in congenital heart disease becomes one of the foremost challenges in the care of CHD patients.

Radiofrequency ablation (RFA) has emerged as a preferred therapeutic modality over long-term antiarrhythmic drugs (AADs) treatment in addressing SVT in CHD patients. This preference is attributed to its superior efficacy and the potential to obviate the need for ongoing pharmacotherapy. Despite the considerable anatomical distortions often present in these patients and the potential challenges related to vascular access, especially in cases involving Fontan procedures, the compelling advantages of transcatheter ablation far outweigh alternative therapeutic options. The procedure's ability to offer a lasting solution to SVT in CHD patients makes it an exceptionally ap-pealing and promising approach in the management of these complex arrhythmias.

In essence, SVT presents a multifaceted challenge within the realm of CHD, but the evolving landscape of therapeutic options, with RFA at the forefront, provides renewed hope for better outcomes and enhanced quality of life for this unique patient population.

## 2 Case description

We present the case of a 21-year-old patient with complex congenital heart disease who was referred to our clinic due to a prolonged history of symptomatic and poorly tolerated recurrent supraventricular tachyarrhythmias, despite receiving chronic treatment with antiarrhythmic drugs (AADs) (amiodarone and bisoprolol).

### 2.1 Medical history

**Figure 1:**
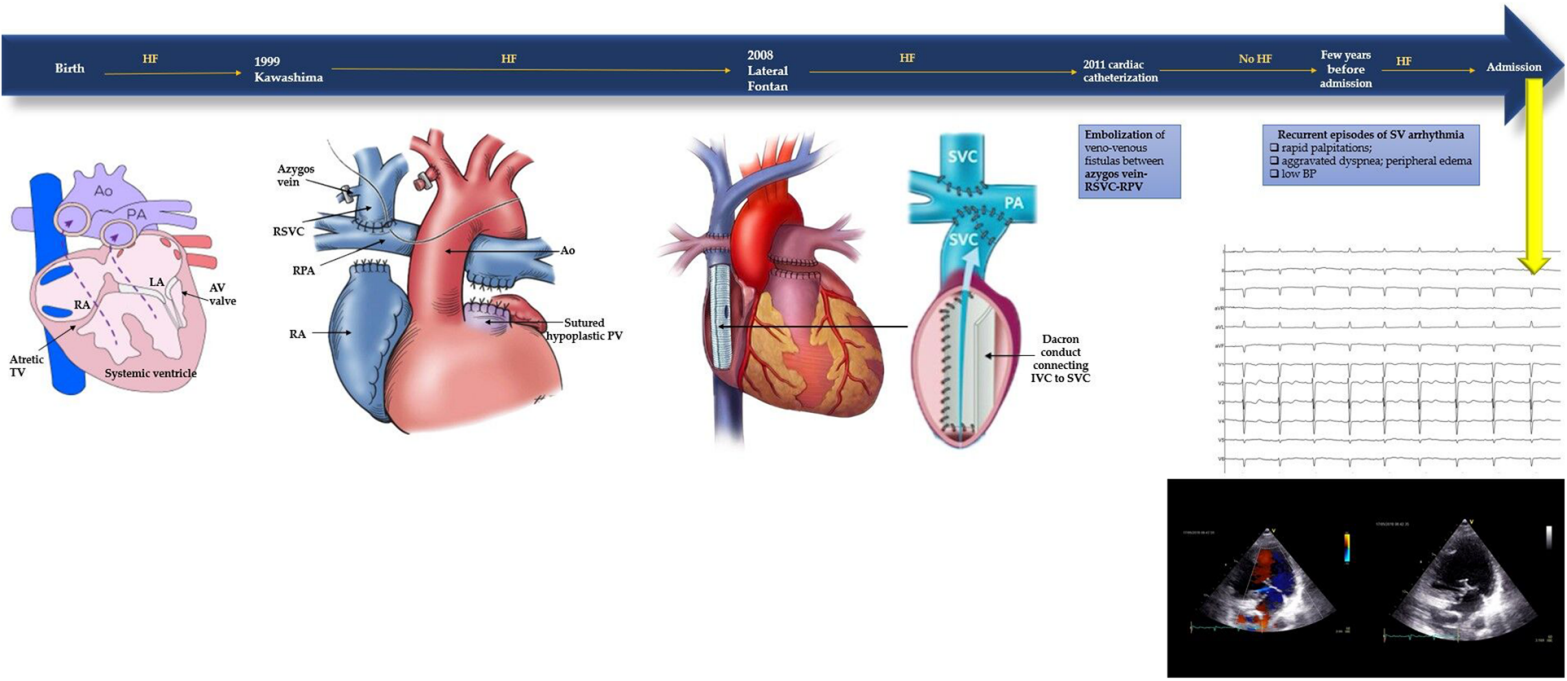
Timeline of the patient's medical history with corrective surgeries (Kawashima procedure, lateral Fontan procedure, cardiac catheterisation) and current symptoms. At admission baseline ECG and TTE were performed. Baseline electrocardiogram: sinus rhythm with slightly wide QRS complex suggestive of non-specific intraventricular conduction delay, left anterior fascicular block and diffuse negative T waves. Transthoracic echocardiography: one ventricle with conserved systolic function, 50%–55% visually estimated; minimal regurgitation of the atrioventricular valve. Apparently only one atrium (the interatrial septum was not visualized); inside of it was present the venous conduct with laminarly flux. It was also viewed the rudiment of the right ventricle. The aorta had complete dextroposition and was slightly more anterior than the pulmonary artery. The aortic valve was tricuspid, with no significant regurgitation. At the left of the aorta, it was also visible the pulmonary artery with a hypoplastic pulmonary valve.

This patient's medical history is characterized by a series of complex palliative surgeries performed during childhood to address a congenital heart condition encompassing tricuspid valve atresia, single ventricle with dextro-transposition of the great arteries, persistent left superior vena cava, and interrupted inferior vena cava. Notably, at the age of one, the patient underwent a Kawashima procedure, which involved creating an end-to-side anastomosis between superior vena cava and ipsilateral pulmonary artery. Subsequently, ten years later, a lateral Fontan tunnel procedure was undertaken due to indications of heart failure. This procedure involved the placement of a Dacron conduit between the suprahepatic veins and the pulmonary arteries, contributing to improved cardiac function. In 2011, during a cardiac catheterization procedure, multiple fistulas were discovered between the azygos vein, superior vena cava and superior pulmonary vein. These were successfully treated through embolization. Signs of heart failure have reappeared in the last years due to recurrent episodes of rapid and regular palpitations with supraventricular tachyarrhythmia documented (Figure 1).

### 2.2 Patient evaluation

Considering the incessant nature of the tachycardia the patient underwent electrophysiological study and radiofrequency ablation with no prior interruption of antiarrhythmic agents. Prior to the procedure transthoracic echocardiography (Figure 1) and multi-slice computed tomography angiography were performed in order to gain an enhanced comprehension of the patient's anatomy, especially taking into account the history of corrective surgeries. Anatomical reconstruction was crucial for more effective planning of the ablation procedure. In addition, a full set of blood tests, a baseline electrocardiogram and a standard anteroposterior thoracic radiograph were performed.

### 2.3 Intraprocedural aspects

The ablation procedure was performed with local anesthesia. For the electrophysiological study, a decapolar deflectable diagnostic catheter was introduced via the right femoral approach through the inferior vena cava into the intraatrial conduct. It was possible to record and pace the right atrium without the need to puncture the Dacron conduit (Figure 3).

**Figure 2:**
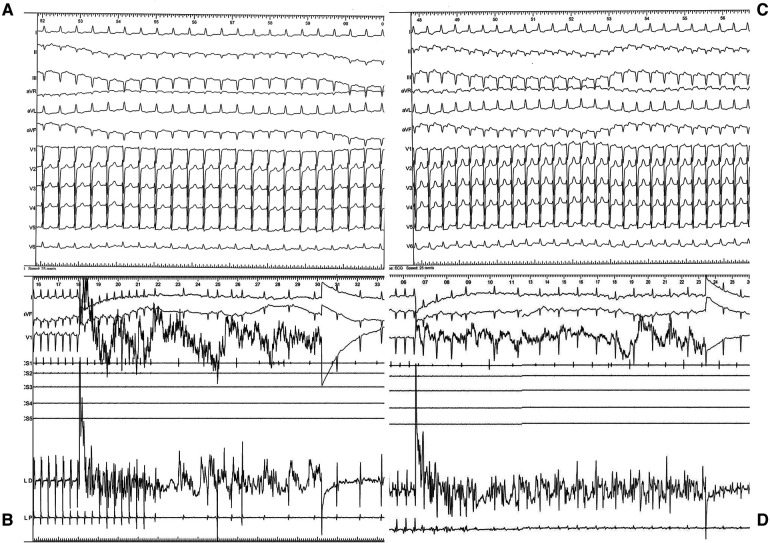
(**A**) ECG during arrhythmia: regular tachycardia, cycle length (CL) of 340 msec, 1:1 ventriculoatrial conduction, RP interval longer than 90 msec, negative P waves in inferior leads, narrow QRS complex without pseudo–R in V1 and pseudo–S in DII–DIII. (**B**) Intraprocedural recording during radiofrequency applications (56 C, 50 W) with arrythmia termination within seconds. (**C**) ECG during second arrhythmia: regular tachycardia, CL of 400 msec, 1:1 ventriculoatrial conduction, long RP interval, positive P waves in inferior leads, narrow QRS complex. (**D**) Intraprocedural recording during radiofrequency applications (56 C, 50 W) with arrythmia termination within seconds.

Diagnostic maneuvers like atrial stimulation were performed at a base cycle of 500 ms with progressive decrease of the extra stimulus from 400 ms resulting in the induction of a supraventricular tachycardia with a 1/1 ventriculoatrial relationship, cycle length of 340 ms, RP'∼120 ms< and negative axis of the P' waves in the inferior leads (Figure 2A). There was no need for isoproterenol administration for arrhythmia induction. Entrainment study confirmed the presence of an orthodromic atrioventricular reentrant tachycardia (ortho-AVRT). Utilizing arterial retrograde transaortic access through right femoral artery, fast anatomical mapping of the left ventricle and left atrium concomitantly with activation mapping of the atrioventricular rings during ortho-AVRT was performed using a Thermocool RMT catheter, a highly flexible mapping and ablation catheter, Stereotaxis remote magnetic navigation and CARTO 3 electroanatomic mapping system. Activation mapping identified the earliest site of atrial retrograde activation as postero-septal, associated with a concealed accessory pathway located in the vicinity of the His bundle. Radiofrequency applications at 56 degrees Celsius and 50 Watts promptly terminated the AVRT (Figure 2B), resulting in a block in the retrograde loop in less than 5 s.

Following this, programmed atrial stimulation (500 ms, 400 ms) induced a second supraventricular tachycardia with a 1/1 ventriculoatrial relationship, cycle length of 400 msec RP'∼180 ms < P'R and positive P' waves in the inferior leads (Figure 2C), and entrainment studies again confirmed ortho-AVRT. A new map of activation during this second tachyarrhythmia was created. This time, the initial site of atrial retrograde activation was antero-septal, associated with a second concealed accessory pathway, also near the His bundle. Radiofrequency applications at 56 degrees Celsius and 50 Watts successfully terminated this AVRT (Figure 2D). At the end of the procedure, there were no other inducible tachyarrhythmias.

**Figure 3:**
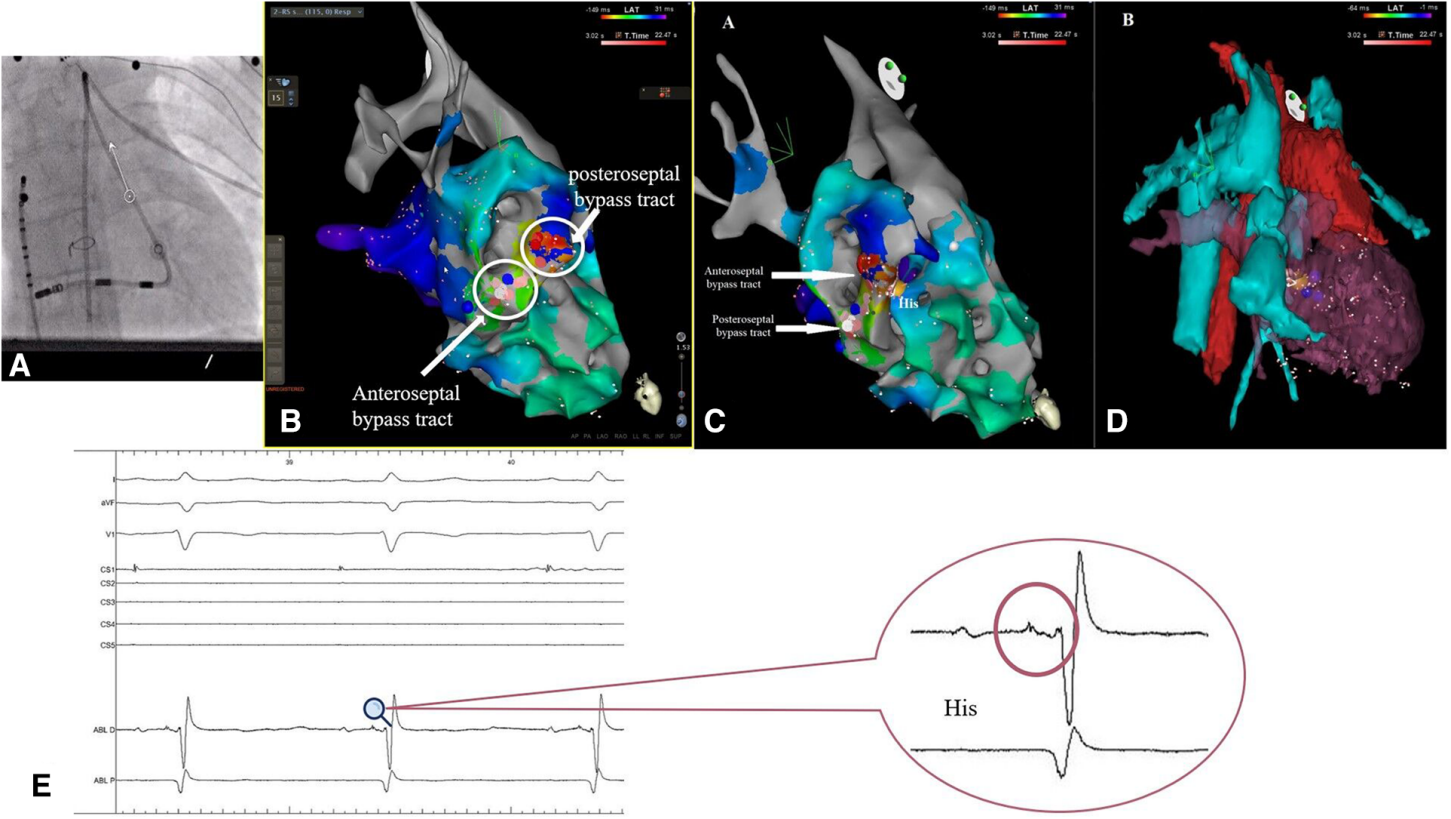
(**A**) Fluoroscopic imagine showing the position of the decapolar deflectable catheter into the intra-atrial conduct, with no puncture of the dacron conduit and the position of the thermocool RMT catheter (**B,C**) electroanatomical CARTO 3 map showing the position of the two accessory pathways in relation to the His bundle (yellow dot). (**D**) Electroanatomical reconstruction using computed tomography. (**E**) Intraprocedural electrogram of the His bundle.

Even upon a comparison of the surface ECGs of these two arrhythmias, a distinct difference was noticeable in P-waves morphology, suggesting the existence of multiple accessory pathways or, at the very least, multiple atrial insertion points. The electroanatomical CARTO 3 maps provided further substantiation of this hypothesis by revealing the existence of two accessory pathways, both situated in close proximity to the atretic tricuspid valve and the His bundle (Figure 3).

### 2.4 Follow-up

Following the procedure, the patient was discharged within 24 h with the recommendation to discontinue the antiarrhythmic medication. Subsequent follow-ups extending beyond 24 months have indicated no recurrence of tachyarrhythmic events, even in the absence of any AADs.

## 3 Patient perspective

Currently at the age of 25, the patient has remained free of arrhythmic events since undergoing radiofrequency ablation, even in the absence of any antiarrhythmic medication during this timeframe. Under optimal medical treatment for preserved ejection fraction heart failure, the patient has maintained a functional NYHA II class of heart failure with no need for hospitalization. Regular follow-up is assessed by means of transthoracic echocardiography, ECG Holter monitoring and blood work by local cardiologist.

## 4 Discussions

The term “Single ventricle” refers to a category of uncommon and intricate congenital heart diseases, characterized by the predominant entry of pulmonary and systemic venous return into a single functional ventricular chamber (3). The overall prevalence is approximately 2 per 10,000 live births (4).

Supraventricular arrhythmias are prevalent in such cases, contributing significantly to morbidity. Over 50% of individuals who underwent an older atriopulmonary connection Fontan procedure encounter atrial tachyarrhythmias two decades post-surgical intervention (5). While the incidence of atrial arrhythmias is lower with contemporary total cavopulmonary connection Fontan surgeries, they still impact 10%–15% of patients within 15 years of follow-up (6).

Indeed, in more than 50% of patients with severe congenital heart defects who reach adult life the onset of atrial arrhythmias by the time they reach 65 years (7, 8). These arrhythmias typically arise from intra-atrial re-entry around surgical scars or anatomical barriers, such as cavotricuspid isthmus-dependent flutter (9). Additionally, various mechanisms, including focal atrial tachycardia, atrioventricular nodal re-entry, atrioventricular re-entry involving a secondary AV node (10), or atrioventricular re-entry through accessory pathways, may be encountered. The differential diagnosis of these mechanisms can be challenging, even in patients without CHD (11).

The embryological anomalies responsible for congenital heart defects can directly affect the development of the conduction system. This can manifest as a simple displacement of the atrioventricular (AV) node and His bundle away from the typical septal position in Koch's triangle. In some cases, malformations lead to accessory or duplicated AV connections, creating the potential for re-entrant tachyarrhythmias (12).

Bypass tracts are a relatively rare cause of supraventricular arrhythmias in adult patients with complex CHD, especially in those with several surgical procedures. Their presence increases the risk of supraventricular tachyarrhythmias, antidromic re-entry, high ventricular rate during atrial fibrillation and even sustained ventricular arrhythmias, leading to a high risk of sudden cardiac death, symptoms of heart failure, palliative procedure failure and debilitating symptoms (13). They are mostly associated with conditions like Ebstein anomaly, congenitally corrected transposition of the great arteries, but there have been reported also cases of tricuspid atresia and bypass tracts, mostly surgically created (14–16).

The prevalence and underlying mechanisms of these arrhythmias can differ based on factors such as age, unique conduction system characteristics, hemodynamic stress resulting from cardiac defects, postoperative complications following surgical procedures (2, 7, 8), as well as acquired heart conditions and associated risk factors (10, 17). In their extensive analysis involving over 38,000 patients with adult congenital heart disease (ACHD) in Quebec, Bouchardy et al. (7) discovered that individuals with a previous atrial arrhythmia history faced a 50% higher mortality rate, a 100% increased risk of stroke or heart failure, and a substantial 300% rise in the likelihood of cardiac interventions compared to those without a history of atrial arrhythmia.

Even supraventricular tachyarrhythmias with relatively slow cycle lengths can be conducted to the ventricle in a 1:1 fashion, resulting in poor tolerance (4, 18). Elevated rates may contribute to the exacerbation of atrioventricular valve regurgitation, thrombus formation within the Fontan pathway, heart failure, syncope, and also sudden cardiac death (9). Management of SVT is a major challenge in the management of adult CHD patients. A comprehensive approach to these patients is crucial, involving multi-disciplinary teams comprising cardiovascular surgeons, cardiologists and electrophysiologists specifically trained in managing CHD patients. This multi-disciplinary approach helps in selecting the best treatment options, increasing success rates, and reducing complications (9).

Especially in Fontan patients, SVTs are frequently resistant to anti-arrhythmic agents. Given the fragile nature of Fontan physiology, patients with sustained supraventricular tachyarrhythmias should be referred for radiofrequency ablation after ruling out underlying conditions, such as cavitary thrombosis (9).

Thoughtful preparation for catheter ablation procedures in patients with congenital heart disease (CHD) requires the formulation of a precise strategy outlining the approach to access the targeted cardiac chamber and address the arrhythmic substrate (19). Catheter ablation in patients with Fontan conduct presents challenges due to distorted anatomic landmarks, limited vascular access (20), suture lines, difﬁcult location of the conduction system, and the existence of multiple mechanism/ circuits of the arrhythmias (1, 21) among other peculiarities (22).

Different approaches can be used to reach the arrhythmogenic area, such as retrograde access to the subaortic ventricle chamber or anterograde access to the pulmonary venous atrium using either a fenestration, trans-baffle or trans-conduit puncture in order to access the atrium. In cases where Cavo-atrial overlap is present enough in case of extracardiac conduit Fontan, a trans-caval puncture, either through hepatic vein or inferior vena cava adjacent to the atrial wall may be feasible (23).

In cases of venous occlusion where vascular access is not possible, the transthoracic percutaneous route may be an option in reaching the atrium and thus be able to map and ablate (24). Rarely ablation procedures can be performed through atrial conducts (22). Nevertheless, in our case, despite the modified anatomy and several palliative surgeries the procedure was performed with the diagnostic catheter advanced in the Dacron conduit, without needing to pass through the conduct.

The strategies for catheter ablation of atrial arrhythmias in Fontan patients have advanced, incorporating newer technologies like high-density multi-electrode mapping catheters, magnetic navigation, irrigated contact force ablation catheters, and the integration of advanced imaging data. These innovations have enhanced the efficiency of ablation procedures in this complex population. Success rates for catheter ablation in Fontan patients vary, with reported ranges between 40% and 75% (20).

A comprehensive understanding of the arrhythmic substrate and mechanism is essential for a successful ablation. Challenges of supraventricular tachycardias mapping and ablation in CHD patients include modified anatomy, which can make quite difficult to access the heart chambers, to identify the critical parts of the arrhythmia circuit, and to create enough ablation lesions in order to successfully eliminate the substrate. Recent innovations in mapping and ablation technology have successfully addressed these issues (25). Nowadays, three-dimensional mapping systems have become a standard component of complex ablation procedures, alongside conventional mapping techniques. Voltage maps contribute to the understanding of scar area distribution, while activation maps assist in visualizing the arrhythmia circuit.

Magnetic resonance imaging, multi-slice computed tomography, intravascular echocardiography and intra-procedural angiography may further enhance understanding of the patient's particular anatomy. Multimodality imaging can be integrated into the map, aiding in understanding the individual patient's anatomy. This not only helps with anatomic orientation but also reduces the need for fluoroscopy (25). Remote magnetic navigation in combination with image integration, has reduced the fluoroscopy exposure, even in extremely complex anatomy cases (26, 27).

Creating effective and secure catheter ablation lesions in CHD adult patients presents several challenges. Persistent congenital heart defects often result in myocardial thickening and dilation (28). For instance, patients who have undergone Fontan surgery may have atrial wall thicknesses of up to 10 mm (29), impeding the establishment of transmural lesions. In significantly dilated cardiac chambers with low blood flow, conventional catheters may encounter limitation in electrode tip cooling, affecting energy delivery during temperature-guided ablation (30).

Studies, such as those conducted by Blaufox et al. (31), have demonstrated that irrigated RFA offers substantially higher power delivery compared to conventional RF ablation. Moreover, the use of irrigated tip catheters has been linked to a reduced number of applications needed for a successful endpoint (13 vs. 23, *P* < 0.01) and higher acute success rates (66% vs. 33%, *P* = 0.019) as shown by Tanner et al. (32). An additional advantage of irrigated RF ablation is the capacity to lower the incidence of thromboembolic complications (28). During RF ablation, the rise in temperature at the tissue-electrode interface can lead to denaturation of serum proteins and tissue charring, thus resulting in coagulum formation at the catheter tip. Active tip-electrode cooling minimizes the incidence of thrombus and char formation at the tip of the ablation catheter (28).

Traditional indicators such as tactile feedback, catheter motion on fluoroscopy and amplitude of the intracavitary electrograms often provide inadequate predictions of tissue contact and lesion formation. Enhancing electrode-tissue contact allows a better transfer of thermal energy to the tissue. Lesion dimensions increase proportionally with contact force (33). Contact force sensor enable the optimization of RF energy delivery while ensuring safety by preventing the excessive force application and also providing directional force vector information, valuable in case of complex anatomy (34).

## 5 Conclusions

This case highlights the pivotal role of transcatheter ablation as a preferred choice over long-term pharmacological therapy in the management of supraventricular tachyarrhythmias in adults with congenital heart disease. It demonstrates the potential benefits of advanced technologies such as highly flexible mapping and ablation catheter, remote magnetic navigation and electroanatomic mapping systems in improving patient outcomes and enhancing their quality of life. Nevertheless, it is essential to acknowledge that each congenital heart disease patient presents unique challenges and requires a tailored approach, showcasing the ongoing need for innovation and expertise in this specialized field.

## Data Availability

The original contributions presented in the study are included in the article/Supplementary Material, further inquiries can be directed to the corresponding author/s.
